# Innovative work behavior of managers: Implications regarding stressful challenges of modernized public- and private-sector organizations

**DOI:** 10.4103/0972-6748.62269

**Published:** 2009

**Authors:** Sudeshna Basu Mukherjee, Anjali Ray

**Affiliations:** Department of Sociology, University of Calcutta, Kolkata, India; 1 Department of Applied Psychology, University of Kolkata, India

**Keywords:** Innovative challenges, Innovative work behavior and managers, Stressful life events

## Abstract

**Background:**

The present study was firstly aimed to find out the nature of stressful life events arising out of the innovative challenges in modernized organizations; and secondly, it tried to identify the relationship between innovative work behavior of managers and the levels of stress arising out of stressful events in modernized organizations (public and private) in West Bengal.

**Materials and Methods:**

Data was collected from a sample of 200 managers, by using 3 tools (General Information Schedule, Life Event Inventory and Innovative Work Behavior Scale) through a face-to-face interview. Responses were subjected to both quantitative and qualitative analyses. The data was statistically treated for ‘*t*’ and ANOVA.

**Results:**

Data highlighted the fact that the qualitative profile of stressful events in the lives of managers expressed specificity in terms of their organizational type (public- and private-sector modernized organizations), and levels of stress from stressful life events were significantly higher among the modernized private-sector managers than those among public-sector managers. The prevalence of innovative work behavior was moderately higher among managers of private-sector modernized organizations than their counterparts in public-sector organizations. The trends of innovative work behavior of the managers indicated much variability due to interaction of their level of perceived stressful challenges for innovation and the global forces of change that have unleashed dynamic, systematic and higher expectation level from them.

Modernization, the act of development, a change for the contingent environment, is the necessity for organizational survival in the organizational system today. It is almost synonymous with change in structure, process and agency. Such change is sometimes radical and sometimes incremental, but under both circumstances managers must develop processes to encourage and guide the changes taking place and create a source of innovation for managing and accomplishing the accompanied variations in the task for survival (Daft, 2004). Thus an organization that has adopted the methods and tasks of modernization has implemented new or unique ways of accomplishing the tasks involving hyper-competition and speed. Innovative change is associated with implementation of technological development amidst challenges of innovative work behavior, along with the focus on team-based functioning. Versatility, communication system, strategic alliances, all designed to maximize the added value, are fast becoming the competitive weapons of the future; and to survive in the fast-evolving organizational world, managers must adopt strategies that realistically reflect their ability to cope with the innovative and multiple future scenarios.

To continuously compete and incrementally design and redesign jobs by promoting and implementing innovations, enhancement of job responsibilities is necessary, and this entails a broad range of new types of job demands and multi-skilling cross-functional skills and cross-functional exposure (O’Driscoll and Cooper, 1996; Gowing *et al*., 1997; Murphy, 1989; Keenan *et al*., 1985; Murphy, 1999). These innovative trends and potentials for beneficial effects in terms of great variations and flexibility have helped to bridge global gaps and increase efficiency and hence modernize organizations, but have also an increased risk of stress due to work overload, pace of work, inter-role conflicts, work-family imbalance and lack of time for rest and recovery (Lundberg, 2000). This type of deviation from the normal functioning or lack of fit arising out of the interaction of people and their jobs is termed as occupational stress by many researchers (Beehr and Newman, 1978). The sources of such stress may be organizational, extra-organizational, individual or group related (Luthans, 2004).

Under such occupational stress arising out of technological advancement and global marketing policy of modernization, managers are required to work under increased pace of managing and handling multitasks pertaining to a technological innovation (new technical artifacts, devices or products), a process innovation (new services, programs, products or procedures) or an administrative innovation (new institutional policies, structures or systems), and this is creating new and increased work pressure (Zaltman *et al*., 1973). Such global competition has forced managers in organizations to become innovative by generating ideas, promoting ideas that are generated and implementing the ideas that have been generated and are likely to get good responses when implemented. Thus such work behavior for innovation is designated innovative work behavior (Janssen, 2000; Basu Mukherjee, 2008). It is a compulsive achievement bestowed upon an individual worker (manager), and the workload evidently created by the displacement of the status quo creates stress for some people who cannot accomplish the demand and those who do adjust with the demands through innovative managerial interventions and expect that the extra workload will be rewarding for their ability. The social exchange theory (Blau, 1964) designates innovative response to job demand of managers as being regulated by perception of effort-reward balance; whereas when an employee or manager is unable to fit into a balance of effort-job-reward, then a conflict leading to stress intensifies. Hence the impact of globalization with its technological, procedural and administrative changes has given rise to innovative challenges for managers to perceive, innovate and cope with changes that are inevitable institutional attributes in the changed scenario (Daft, 2004; McShane, 2001).

Realizing the importance of innovative work behavior of managers in the process of initiation of climate of innovation in modernized organizations for organizational development, and anticipating their health risk and psychosocial cost of stress for managers in the face of innovative challenges, the present researcher was interested to measure the perceived life-events stress in the innovative process and the pattern of innovative work behavior in modernized organizations. As a step in this direction, a research proposal was framed involving the managers of private- and public-sector modernized organizations with the following objectives in mind:

- To study the stressful events in the lives of managers in the face of innovative challenges of modernized private and public organizations.
- To study the stressful events in the lives of managers of modernized organizations with respect to their rank/position (senior or junior).
- To study the nature of innovative work behavior expected of the managers in modernized public- and private-sector organizations with respect to the levels of stress involved (high or low).

Contextually, some of the items of the Life Event Inventory were specifically introduced, as they were situational and occupationally related to contingent structural and procedural affairs of a modernized organization.

## MATERIALS AND METHODS

### Variables

#### Life-events stress

Life-events stress is stress developing out of situational encounters that are scheduled and unscheduled events or desirable or undesirable events; and job-typical and profession-specific situations or service conditions, etc., were considered as occupational stressful life events (Choudhury *et al*., 2005; Pestonjee, 1999). The job-typical life event stress evolving from technological progress, demands of changing technical skills and/or knowledge, management communication and information feedback systems, working situations, innovativeness, social conditions of work and personal life of executives was considered as measured variable of the study (Bhattacharya *et al*., 2004; Hammer *et al*., 2004; Horan, 2002; Roy and Basu Mukherjee, 2006).

#### Innovative work behavior

Innovation is the process of creating new ideas and putting them into practice. In this study, innovative work behavior of the executives was defined as the self-reported level of different behavioral tasks, namely, idea generation, idea promotion and idea realization. The job-related components were recognized as important personal-level factors related to innovation in the workplace (Amabile *et al*., 1996; Kanter, 1988; Scott and Bruce, 1994; Basu Mukherjee, 2008).

### Tools

1. General Information Schedule with the job-demo graphic and socio-demographic information.
2. Life Event Inventory: This was a specifically developed inventory by Basu Mukherjee and Roy (2006) for managers of modernized organizations in Indian situations. The inventory consisted of 35 items covering 6 components. The textural concepts or domains were as follows: Innovative changes, interpersonal communication, management, work situation and impact of modernization and personal attributes both in work life and family (personal factors and extra-organizational factors). The item total correlation of schedule ranged between 0.39 and 0.74, and component correlation ranged between 0.68 and 0.86. The result with regard to test-retest reliability of the inventory was 0.79.
3. Innovative Work Behavior Scale: This was a 9-item scale originally developed by Janssen (2000), and it was locally adapted by the investigators. This scale consists of 3 components: Idea generation, idea promotion and idea implementation. The reliability of the original scale was 0.85, and the reliability of the locally adapted version of the scale was high (Spearman Brown-‘*r*’ = 0.84 and Cronbach’s Alpha-‘*r*’ = 0.95). The item total correlation of the locally adapted version ranged from 0.44 to 0.78.

### Sample

- Forty modernized organizations (20 public and 20 private) were selected on the basis of the criteria that were the necessary requirements for an organization to be called innovatively modernized.
  - They fulfilled the ISO standards.
  - They have had a de-layering/downsizing exercise after the 1991 liberation policy (restructuring of the organization).
  - They followed at least two among the following procedural systems:
    Competency mapping/performance appraisal, human resource audit, business balance scorecard, benchmarking, KSAO (knowledge, skill, ability, others), Kaizen (continuous improvement).
- Altogether, 200 managers equiproportionately drawn from public and private organizations. The sample was also equiproportionately drawn with regard to ranks/positions (senior or junior) according to designations as per organizational allotments through purposive sampling based on the inclusion criteria of age range (30-40 years) and number of years of service (5-10 years).

### Data collection

Responses of 200 managers of private- and public-sector organizations were collected and considered through the General Information Schedule, Life Event Inventory, and Innovative Work Behavior Scale.

### Statistical methods

Percentage, mean, standard deviation, ‘*t*’ and ANOVA were calculated on the basis of the scores of the above-mentioned inventories and scale.

## RESULTS AND INTERPRETATION

### The trends of stressful life events

The Life Event Inventory (LEI) responses as collected were treated for a) quantitative analysis and b) qualitative analysis for description of pattern of stressful events for the groups of managers. In order to test the significance of difference between managers (junior and senior in accordance with the existing norms of respective organizations) of modernized public and private organizations with respect to their levels of influences of perceived stressful events, the responses of LEI were processed for two-way ANOVA, and the results of ANOVA are presented in Table 1. The mean and the significant F-ratio values [Table 1] of Life Event Inventory scores revealed that the overall perceived strength of influence of perceived job-specific stressful life events was significantly higher (M-17.69 and F-9.43) among the modernized private-sector managers than those of public-sector managers. The nature of such feeling also projected marked specificity (F-4.57) with respect to the ranks/ positions of the managers (junior and senior). Further, such trends of differences in the life events of managers in the two types of organizations (public and private) also indicated variability due to interaction of the ranks/positions of managers (F = 8.27).

**Table 1:** Mean and mean difference and ANOVA results for life event inventory responses of managers with respect to their type of organization (public or private) and ranks/positions (junior or senior)

Variable	Mean values and mean differences on LEI scores for			F-ratio for sources of variations		
	Overall sample	Managers of two types of organizations (public and private)	Managers in rank/position (junior and senior)	Within two types of organizations (A)	Within two rank conditions of managers (B)	Due to interactions of types of organizations with rank position (A and B)
Life event	M- 16.90 SD-3.81	Pub-M-16.11 Pvt-M-17.69 M D-1.34	Jun-M-16.35 Sen-M-17.45 M D-1.10	943*	4 57*	8.27*

* indicates the ‘F’ value significance at 0.05 level

A qualitative analysis for description of pattern of stressful events for the two groups (public and private) of managers was carried out on the basis of percentage of responses. Considering ‘*t*’ test results, the profile of significant life events as designated by the specific groups is displayed in Table 2.

**Table 2:** Significant differences in the qualitative profile of most prominent life events as mentioned by at least 55% of the respondents in both groups of managers

List of significant life events and ‘*t*’ values				
Components (LEI)	Private-sector managers		Public-sector managers	
	List of significant life events	‘*t*’ value	List of significant life events	‘*t*’ value
Innovative change	Pressure of high expectations and achievement desire in organizations	5.46	Implementation of new standard tools of quality and environment	6.96
	Demand of continuous expectation of innovativeness	5.00	Pressure for continual improvement to increase efficiency	5.17
	Generation of enterprise resource planning	4.18	Dealing with new technology	5.84
	Pressure of maintenance of benchmarking (creation of reliable performance baseline)	4.27		5.17
	Re-occurring unscheduled demands	5.46		3.87
	Downsizing and de-layering	4.74		
Interpersonal communication	Lack of socially supportive work environment	4.78		
Management	Demands of quality performance/response on time	4.67	HR Audit	5.24
	Imbibing the culture of team-based work	4.24		
	Regular competency-mapping exercises	4.67		
Work situation	Regular overstepping of scheduled working hours	4.27	Lack of comfortable infrastructure/ facility for work	5.24
Personal factors	Work-family balance/family-work balance	4.74	Unsatisfactory career advancement opportunity	4.78
	Anxiety of job security	5.17	Trouble with work implementation at practical level	4.64
Extra-organizational forces	Deprivation of time for family life commitments	5.24	Outsourcing of jobs	4.18
	Restrictive autonomy due to bureaucratization and for utilization of leadership qualities	5.16	Financial crisis management	3.44

Significant ‘*t*’ values of Table 2 highlighted 9 events covering 3 stress-generating events: a) innovative change in the form of pressure to implement new standard tools of quality and environment (*t*-6.96), demand of continuous improvement to increase efficiency (*t*-5.17) and dealing with new technology (*t*-5.84), b) management, such as the aspect of human resource audit (*t*-5.24), c) work situation, like lack of comfortable infrastructure/facility in their work (*t*-5.24) and d) events related to personal factors and extra-organizational forces — unsatisfactory career-advancement opportunity (*t*-4.78), trouble with work implementation at the practical level (*t*-4.64), outsourcing of jobs (*t*-4.18) and financial crisis management (*t*-4.63) were more stress-generating events for public-sector executives.

On the other hand, the private-sector executives found 15 events covering domains of stressful events due to a) innovative change — pressure of high expectations and desire for achievements in organizations (*t*-5.46), demand of continuous innovativeness (*t*-5.00), generation of enterprise resource planning (*t*-4.18), pressure of maintenance of benchmarking (*t*-4.27), re-occurring unscheduled demands (*t*-5.46) and downsizing and de-layering of organizations (4.47), b) problems of interpersonal communication — due to absence of socially supportive work environment (*t*-4.78), c) events related to management demands of quality performance and response on time (*t*-4.67), imbibing the culture of team-based work (*t*-4.24) and regular competency-mapping exercise (*t*-4.67), d) undesirable work situation due to working beyond scheduled working hours (*t*-4.27) and e) the strains of events related to personal factors and extra-organizational forces - due to work-family imbalance (*t*-4.74), anxiety of job security (*t*-5.17), deprivation of time to fulfill family life commitments (*t*-5.24) and restrictive autonomy due to bureaucratization and leadership quality (*t*-5.16) were stressful for private-sector executives.

The study highlighted that both public- and private-sector managers differed significantly with respect to the quality of stressful life events. It was observed that these events imposed cumulative strain on employees that generated stress, and prolonged impact of such stressful events damaged the self-esteem and performance quality, which indirectly hindered the managerial activities and innovative work behavior of respective groups of managers (public and private) to enable them to cope with the demands of innovative changes of modernized organizations (Bhattacharya *et al*., 2004; Choudhury *et al*., 2005; Roy and Basu Mukherjee, 2006; Fuller *et al*., 2003; Iwasaki, 2001; Melamed *et al*., 1996).

### Innovative work behavior

Table 3 supportively highlighted that the overall level of innovative work behavior of the managers of modernized organizations was moderately high (M =40.67). The results indicated that the level of innovative work behavior of private-sector managers was significantly higher (M-42.70, F-6.33) in the organization than their counterparts in the public sector because their job depended on their performance. Such innovative work behavior was an adaptive competence that was necessary for the organizational survival in the global market.

The trend of such innovative work behavior rests on disciplined management of idea inception, furtherance and realization. Reasons for the level of innovative work behavior being moderately high among private-sector managers may be “system thinking” (Senge, 1990). System thinking is the ability to understand the complex causal relationship among a set of organizational factors and issues. It is a framework for seeing interrelationship rather than things in pieces.

**Table 3:** Mean standard deviation mean difference and ANOVA results for innovative work behavior of managers in modernized organizations (public and private organizations with high and low levels of stress)

Variable	Mean (M), standard deviation (SD) and mean difference (MD) for innovative work behavior scale scores for executives in			F-Ratio values for sources of variation		
Innovative work behavior components	Sample scores	Two kinds of organizations (public and private)	Two groups of executives (with high and low levels of stress)	Within both types of organizations (A)	Among executives with two levels of stress (B)	Due to interaction between kinds of organization and levels of stress (A and B)
Idea generation	M-1335	Pub-M-13.16	LS-12.86	0.778	2.39	6.71*
	SD-4.50	Pvt-M-13.5 }	HS-13.78			
		MD-0.38	MD-0.92			
Idea promotion	M-13.48	Pub-M-12.86	LS-13.44	5.03*	0.005	11.21*
	SD-4.41	Pvt-M-14.10	HS-13.52			
		MD-1.24	MD-0.08			
Idea implementation	M-13.84	Pub-M- 2.62	LS-13.85	13.06*	0.131	11.98*
	SD-5.34	Pvt-M-15.06	HS-13.81			
		MD-2.44	MD-0.46			
Innovative behavior	M-40.67	Pub-M-36.64	LS-41.08	6.33*	0.502	11.74*
	SD-13.12s	Pvt-M-42.70	HS-40.20			
		MD-6.06	MD-0.88			

* indicates the ‘F’ value significance at 0.05 levels. LS - Low levels of stress; HS - High levels of stress

Component-wise analysis highlighted that the private-sector managers of modernized organizations were more successful in promoting (M-14.10, F-5.03) the trends of percolation of innovative ideas to superiors and subordinates as well as implementing (M-15.06, F-13.06) for realization of the goals and objectives of the innovative ideas in modernized organizations than the public-sector managers. Results revealed that private-sector managers had better opportunities for contingent thinking; they had space to delineate all the innovative contextual factors that could influence a good practice or process for survival. Important contingencies involving anything from innovative business strategies or culture to innovative external business challenges were acceptable for survival in the global market. Yet both the private- and public-sector managers were on similar platforms with regard to idea generation (M-13.54 and M-13.16), a creative aptitude for production of novel and useful ideas in any domain, to meet work-related problems, incongruities, discontinuities and emerging trends of organizational demands. Such outcomes were projected in the research findings by various researchers(ie, Amabile, 1988; Baer *et al*., 2003; Janssen, 2003; Janssen, 2004; and Thornquvist, 2007). Results showed that such trends indicated much variability due to interaction of the level of perceived stressful innovative challenges by the managers.

Such discrepancy between private- and public-sector managers may be attributed to their (private-sector managers’) relatively higher level of competitiveness and pre-entry professional multi-skilled training for the job competencies [Table 4], combined with the impact of energetic drive and risk-taking motivation, which act as impetus for promotion of activities in the face of new challenges of innovation and lack of job security; as compared to the public-sector managers, who have job security and for whom promotion is not performance based. Present findings about relatively better trends of innovative work behavior of the managers are supported by Cassar *et al*., (2005); Janssen *et al*., (2004); Stahl and Steeger, (1997); and Andrews and Farris, (1972).

**Table 4:** Profile of the background information and self-assessed attributes of the respondent groups of managers and the χ^2^ values

Variables	Components	No. of executives in		χ^2^
		Private-sector organizations	Public-sector organizations	
Educational background	Graduates	44	48	0.08
	Postgraduates	66	62	0.06
	Professional qualifications	62	33	8.85
Professional status	Senior	22	10	4.50
	Junior	42	23	5.55

## CONCLUSION

The challenge of innovation and modernization has increased demands of high work pace, continuous innovation, competition and strategies for coping with new conditions of techno-social challenges, which are indirectly influencing and are influenced by the level of occupational stress of the managers. In this context, the database facts of the study highlighted certain inferences: The life event patterns in the lives of the managers of modernized organizations have changed under the impact of the global question of survival. The number of stressful life events generated by the innovative changes of globalization was more for private-sector managers than the public-sector managers. In the face of challenges of modernization, the managers of the private sector were more innovative in their work behavior than their public-sector counterparts. For them (private-sector managers), survival depends on the ability to utilize transformed technology, by creating and maintaining the onward drives of the highly dynamic work situations. It is imperative, therefore, to project the strategies that realistically reflect their ability to manage multi-layered future embedded in the shift. The trends of differences in the nature of work behavior of private- and public-sector managers showed much specificity with respect to the levels of stress of the managers in their respective job situations. As global competition has increased, managers in organizations have been forced to become innovative, and the stress-coping strategies in the form of innovative work behavior are an important precondition for survival and development of organizations and its managers.
